# Children's Rights

**Published:** 1920-05-29

**Authors:** 


### CHILDREN'S RIGHTS.

### Prospects of a New Health Era.

THE Belfast papers publish accounts of what appears to have been a most useful and successful health exhibition, opened by Lady Shaftesbury. It was organised by the Women's National Health Association in conjunction with the Belfast Council of Social Welfare. The exhibits illustrated many features of health preservation. There were models and plans of dwelling-houses for workers which were shown in the Ideal Homes Exhibition in London recently, and approved by the English Ministry of Health; while another feature was a special housing model from the London County Council, which represents houses at present being built on the Roehampton Estate. A dental section illustrated the care of the teeth; a milk section showed the importance of a clean milk supply; a fly section demonstrated the dangers which arise from insect pests; a food section gave the actual nourishing value of different foods; while the child welfare section was devoted to the requirements for bathing, feeding, clothing, and sleeping of infants. "Thrift" garments made from cast-off clothing were on view, while another section gave information and advice to mothers. There was also an up-to-date literature table dealing with all matters connected with health.

It is most encouraging to all those engaged in health propaganda that exhibitions of this kind, with courses of lectures, are becoming more general and more popular. It has always seemed curious that the care of health should have been for so long an uphill advocacy.

Speaking at the Belfast Exhibition, Dr. E. Coey Bigger (chairman of the Irish Public Health Council) said some of the many factors which prejudicially affected child-life were poverty, ignorance, insanitary houses, food, hereditary disease, and the employment of mothers. They must endeavour to improve the conditions of the children of the poor, so that they would more closely approximate to the conditions of the children of the middle and independent classes. (This is a point which has frequently been made in these columns, but education is just as important.) Preventive medicine was the foundation-stone of modern medicine, and every child had the right to be born under the most favourable conditions, and the best kind of education was the education that helped the mother to help herself. He considered that under proper conditions of housing, sanitation, and careful supervision, together with sufficient and suitable food, the deaths of infants in the city of Belfast could be reduced by at least fifty per cent., or, in other words, out of every two children who died, one could be saved. The health of the child could be supervised from its birth up to five years of age under the child-welfare scheme, and from five years onwards during its school period, under the scheme for the medical inspection and treatment of school-children the Government paying half the cost. The Irish Public Health Council had been, for several months past, engaged in the task of preparing a report for the Irish Government on the Irish medical and health services. The Council had now completed that part of their work, and it was hoped their report would be in the hands of the Government and available for publication very shortly. The object which the Council had in view was to suggest such changes in existing health legislation as would bring about the betterment of general health conditions in Ireland. They had made recommendations of a most comprehensive character, which, if carried into effect, would open up a brighter era in health administration in Ireland and tend to afford for all who were in need of it the best possible treatment.

The analogous Council for Wales has already presented a model scheme (which has been laid on the table of the House of Commons) based on the principle of a medical attendant to every family and providing for a most complete system of treatment, conveyance, and accommodation. The Minister of Health is said to regard it as an ideal to be aimed at ultimately, but too costly to be inaugurated *en bloc*.

